# Synergistic interactions of melatonin and glucocorticoids in alleviating allergic airway inflammation

**DOI:** 10.3389/fmed.2026.1738965

**Published:** 2026-02-25

**Authors:** Zhe Zhang, Jipeng Wang, Ting Ma, Xiuqin Zhang, Guilian Chen, Baolan Wang

**Affiliations:** 1 Physical Examination Center, The Affiliated Huai'an No.1 People's Hospital of Nanjing Medical University, Huai'an, Jiangsu, China; 2 Department of Respiratory Medicine, The Huai'an Clinical College of Xuzhou Medical University, Huai'an, Jiangsu, China; 3 Department of Respiration Medicine, The First Affiliated Hospital of Soochow University, Suzhou, Jiangsu, China

**Keywords:** allergic diseases, asthma, circadian rhythm, glucocorticoids therapy, melatonin

## Abstract

**Background:**

This study investigates the synergistic effects of melatonin and glucocorticoids in the management of asthma and allergic airway inflammation. While glucocorticoids are the cornerstone of asthma treatment, melatonin’s anti-inflammatory properties, potentially mediated through circadian rhythm regulation, may enhance their therapeutic efficacy.

**Methods:**

A case–control clinical study was conducted to assess asthma control scores, biochemical markers, exhaled nitric oxide (FeNO), and sleep quality. Additionally, an animal model was utilized to evaluate the effects of melatonin and dexamethasone combination therapy on airway inflammation and circadian rhythm gene expression.

**Results:**

The clinical study found significant correlations between asthma control scores, melatonin and PSQI sleep quality, suggesting that melatonin play crucial roles in asthma control. In parallel, animal experiments revealed that melatonin reduced allergic airway inflammation and stabilized circadian rhythm gene expression. Furthermore, the combination of melatonin with glucocorticoids exhibited a significantly enhanced effect in reducing allergic airway inflammation.

**Conclusion:**

Melatonin, when combined with corticosteroids, may represent a promising therapeutic strategy for allergic asthma, potentially improving asthma control through modulation of inflammation and circadian rhythm regulation. Further studies are needed to fully elucidate the mechanisms underlying this synergy and to evaluate the long-term therapeutic benefits.

## Graphical abstract

**Figure:**
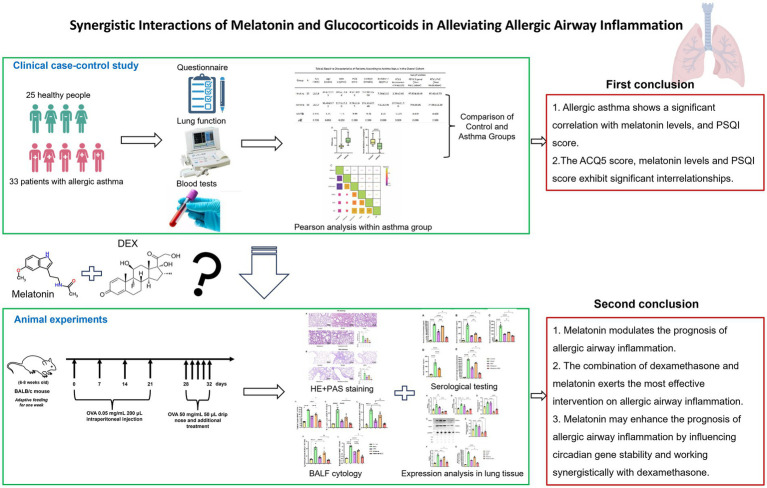

## 1 Introduction

Allergic asthma is a chronic allergic airway condition characterized by airway obstruction and heightened responsiveness, significantly affecting patients’ quality of life (1). The increasing prevalence of allergic asthma underscores the urgent need for effective therapeutic options. While current management strategies, including inhaled corticosteroids and bronchodilators, provide relief for many patients, they are often insufficient for some individuals and may introduce undesirable side effects (2–4). Thus, exploring alternative treatment modalities is essential for enhancing patient outcomes.

Recent studies have suggested that melatonin, a hormone primarily known for its role in regulating circadian rhythms and sleep–wake cycles (5, 6), may also modulate immune responses and airway inflammation associated with allergic asthma (7, 8). Evidence indicates that melatonin could play a critical role in the pathogenesis of asthma and other allergic conditions (7, 9), yet the mechanisms of its action remain poorly understood. Notably, the relationship between melatonin levels, asthma control, and the inflammatory processes involved in asthma exacerbations is not comprehensively characterized.

Current literature indicates that melatonin impacts several biological pathways pertinent to asthma, including the regulation of immune responses and inflammation (10, 11). For example, numerous studies have documented its anti-inflammatory properties (9, 12), suggesting that melatonin may mitigate airway inflammation severity and enhance asthma management. However, the potential synergistic effects of melatonin when combined with corticosteroids in the treatment of allergic asthma warrant further exploration. This study seeks to address the existing research gap by examining the interplay between melatonin and glucocorticoid levels in asthma patients.

To investigate this, we employed a case–control study design, analyzing data from 33 allergic asthma patients and 25 healthy controls. Our methodology includes standardized questionnaire assessments and biological marker evaluations to compare asthma control status and melatonin levels. Furthermore, we utilized a mouse model of allergic airway inflammation to examine the biological effects of melatonin and its interactions with inflammatory mediators and the expression of circadian rhythm-associated CRY1 Genes. By integrating clinical and experimental methodologies, this research aims to elucidate the role of melatonin in asthma pathophysiology and its potential therapeutic implications.

The primary objective of this study is to assess the relationship between melatonin and glucocorticoid levels, sleep quality, and asthma control status. The findings may provide valuable insights into the mechanisms underlying asthma management and contribute to the development of innovative treatment strategies that incorporate melatonin as an adjunct therapy in allergic asthma. This exploration is particularly relevant given the pressing need for effective interventions for patients whose asthma remains inadequately controlled despite conventional therapies.

## 2 Materials and methods

### 2.1 Clinical information

#### 2.1.1 Study population

This study utilized a case–control design. The case group consisted of 33 individuals diagnosed with allergic asthma, as defined by the GINA guidelines (13), who had not received glucocorticoid treatment within the past month. The control group comprised 25 healthy individuals, aged between 18 and 50 years, recruited from routine health check-ups during the same consultation period. Matching between the asthma and control groups was based on gender, age, BMI, education level, and living environment. This study was approved by the Ethics Committee of the Affiliated Huai’an No.1 People’s Hospital of Nanjing Medical University (Permit number: KY-2023-173-01).

#### 2.1.2 Datasets of study subjects

Fasting serum samples were collected from both groups, with approximately 6 mL of blood drawn (in a yellow biochemical tube) before 10 a.m. to measure melatonin, blood eosinophils, IgE, and other relevant biomarkers. Lung function tests, bronchodilator response assessments, and measurements of fractional exhaled nitric oxide (FeNO) were conducted. The Asthma Control Questionnaire (ACQ5) and Pittsburgh Sleep Quality Index (PSQI) scores were obtained through standardized questionnaires.

#### 2.1.3 Sample processing

Approximately 10 mL of blood was drawn from each subject around 10 a.m. daily. Of this, 3 mL was allocated for routine blood tests, while the remaining 7 mL was processed for further analyses. The blood was allowed to clot at room temperature for 30 min, followed by centrifugation at 3000 rpm for 10 min. The resulting serum was separated, aliquoted, and stored at −80 °C. Serum melatonin levels were measured using a Human MT (Melatonin) ELISA Kit (Elabscience, Cat. No. E-EL-H2016), and serum IgE levels were determined using a Human IgE ELISA Kit (Beyotime Biotechnology, Shanghai, China; Cat. No. PI479), according to the manufacturers’ instructions.

### 2.2 Animal experiments

#### 2.2.1 Animals

Female BALB/c mice, aged 8 weeks and weighing 18–20 grams, were obtained from Shanghai BK/KY Biotechnology Co. Ltd. All mice were housed under controlled conditions at a temperature of 20–26 °C, It was provided with free access to food and water, and was subjected to a 12-h cycle of light and darkness. All experimental protocols were approved by the Animal Care and Use Committee at the Affiliated Huai’an No.1 People’s Hospital of Nanjing Medical University (Permit number: DW-P-2023-001-22).

#### 2.2.2 Model of OVA-induced allergic airway inflammation and therapeutic interventions

**Figure 1:**
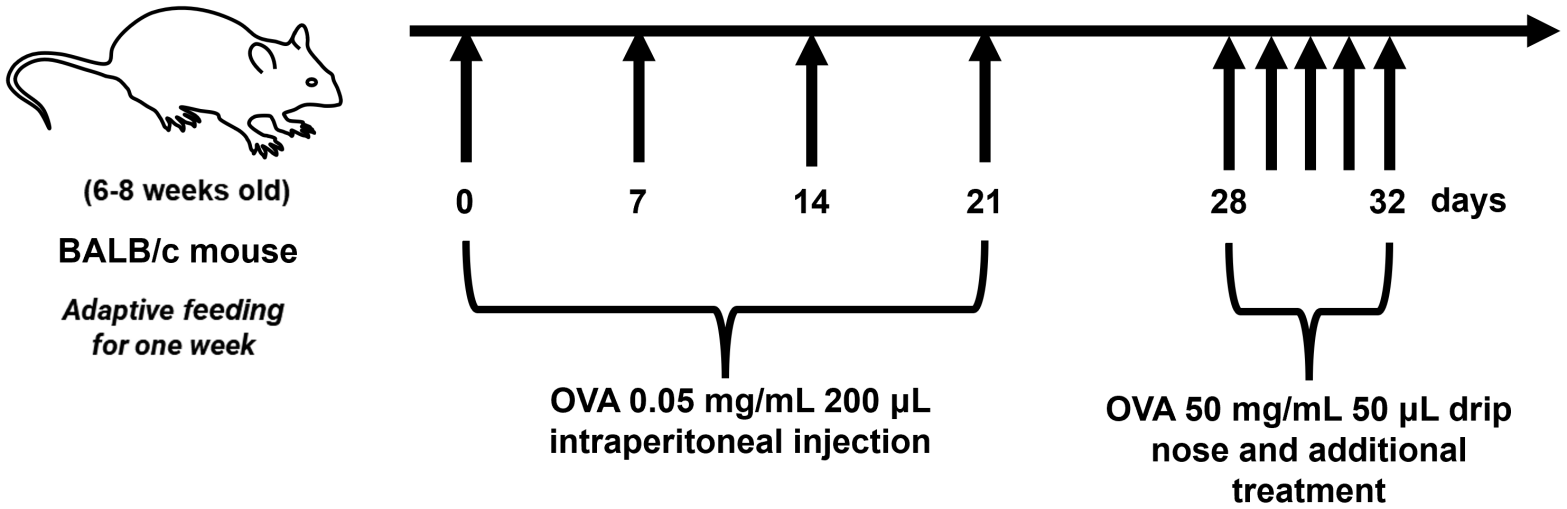
Flowchart of the construction and treatment process for the mouse model of allergic airway inflammation.

After a one-week acclimatization period, the mice were randomly divided into five groups: normal control group (Control; *n* = 6) and model group (Model; *n* = 6), dexamethasone-treated group (DEX; *n* = 6; treated with 3.0 mg/kg dexamethasone), Melatonin-treated group (Melatonin; *n* = 6; treated with 15 mg/kg melatonin), and Melatonin + dexamethasone-treated group (Melatonin + DEX; *n* = 6; treated with melatonin 15 mg/kg and dexamethasone 3.0 mg/kg) (14). Mice in the Model, DEX, Melatonin and Melatonin +DEX groups received intraperitoneal injections of 200 μL of the sensitizer (Sensitizer: Dissolve 0.018 g ovalbumin in 6 mL PBS to make a 3 mg/mL solution, dilute 70 μL with 2.03 mL PBS to 0.1 mg/mL, then mix with 2.1 mL aluminum hydroxide to 0.05 mg/mL) on days 0, 7, 14, and 21. From days 28 to 32, each mouse received a nasal drip of the activator (50 μL/day) between 9:00 and 10:00 p.m. (Activator: Dissolve 0.204 mg ovalbumin in 4.08 mL saline to prepare a 5% ovalbumin solution), and the treatment groups received their respective medications via gavage 1 h after each challenge (15, 16). Mice were euthanized 24 h after the last stimulation (day 33). The entire experimental process is shown in Figure 1.

#### 2.2.3 Specimen processing

##### 2.2.3.1 Collection and analysis of bronchoalveolar lavage fluid (BALF)

Twenty-four hours after the final challenge, mice were sacrificed between 9:00 and 10:00 p.m. The trachea and lungs were gently exposed, and the right lung was ligated. Bronchoalveolar lavage (BAL) was performed on the left lung by instilling 0.3 mL of cold phosphate-buffered saline (PBS) five times. The retrieved BAL fluid (BALF) was then centrifuged at 4 °C for 10 min at 1000 rpm. The cell pellets were re-suspended in 200 μL of PBS for the purpose of performing total cell counts, and a blind cellular differentiation of eosinophils, monocytes, lymphocytes, and neutrophils was carried out using a hemocytometer.

##### 2.2.3.2 Serum

Blood samples were collected via orbital blood sampling and transferred into Eppendorf tubes. Samples were allowed to clot at room temperature for 2 h and were then centrifuged at 3,000 rpm for 15 min. The obtained serum was collected and stored at −20 °C until analysis. Serum levels of immunoglobulin E (IgE), MUC5AC, corticosterone, interleukin (IL)-4, IL-5, and IL-13 were measured using commercially available enzyme-linked immunosorbent assay (ELISA) kits according to the manufacturers’ instructions. Mouse IL-4, IL-5, IL-13, IgE and corticosterone ELISA kits were obtained from Beyotime Biotechnology (Shanghai, China) (Cat. Nos. PI612, PI620, PI539, PI476, and PC100, respectively); the mouse MUC5AC ELISA kit was obtained from Saipuru Bio (Wuhan, China) (Cat. No. SP13899); All samples were assayed in duplicate, and concentrations were calculated from standard curves.

##### 2.2.3.3 Lung tissue

After blood collection, mice were euthanized via cervical dislocation. The lung tissue was excised and washed with physiological saline. The left lung was fixed in a 5 mL Eppendorf tube containing 4% paraformaldehyde for morphological analysis, while the right lung was rapidly frozen in liquid nitrogen for 30 min and subsequently stored at −80 °C for subsequent analysis of gene and protein expression.

##### 2.2.3.4 Morphological analysis of lung tissue

Lung tissue was fixed in 4% paraformaldehyde for 48 h with gentle shaking, followed by dehydration, embedding, sectioning, and staining with hematoxylin and eosin (HE) and Periodic Acid-Schiff (PAS) staining. The tissue damage, as well as positive expression, were assessed using optical microscopy.

### 2.3 Statistics

All data were analyzed using GraphPad Prism 10.0. Quantitative data are presented as mean ± SD. Two-group comparisons were performed using Student’s *t*-test, and multiple-group comparisons were analyzed by one-way ANOVA with appropriate *post hoc* tests. Semi-quantitative data (HE scores, PAS scores) were analyzed using Mann–Whitney U or Kruskal–Wallis tests. All tests were two-tailed, with *p* < 0.05 considered statistically significant. Effect sizes and 95% confidence intervals were calculated where applicable. Statistical significance in figures is indicated as: *p < 0.05, **p < 0.01, ***p < 0.001, ****p < 0.0001, and “ns” for no significant difference.

## 3 Results

### 3.1 Findings from the case–control study

**Figure 2:**
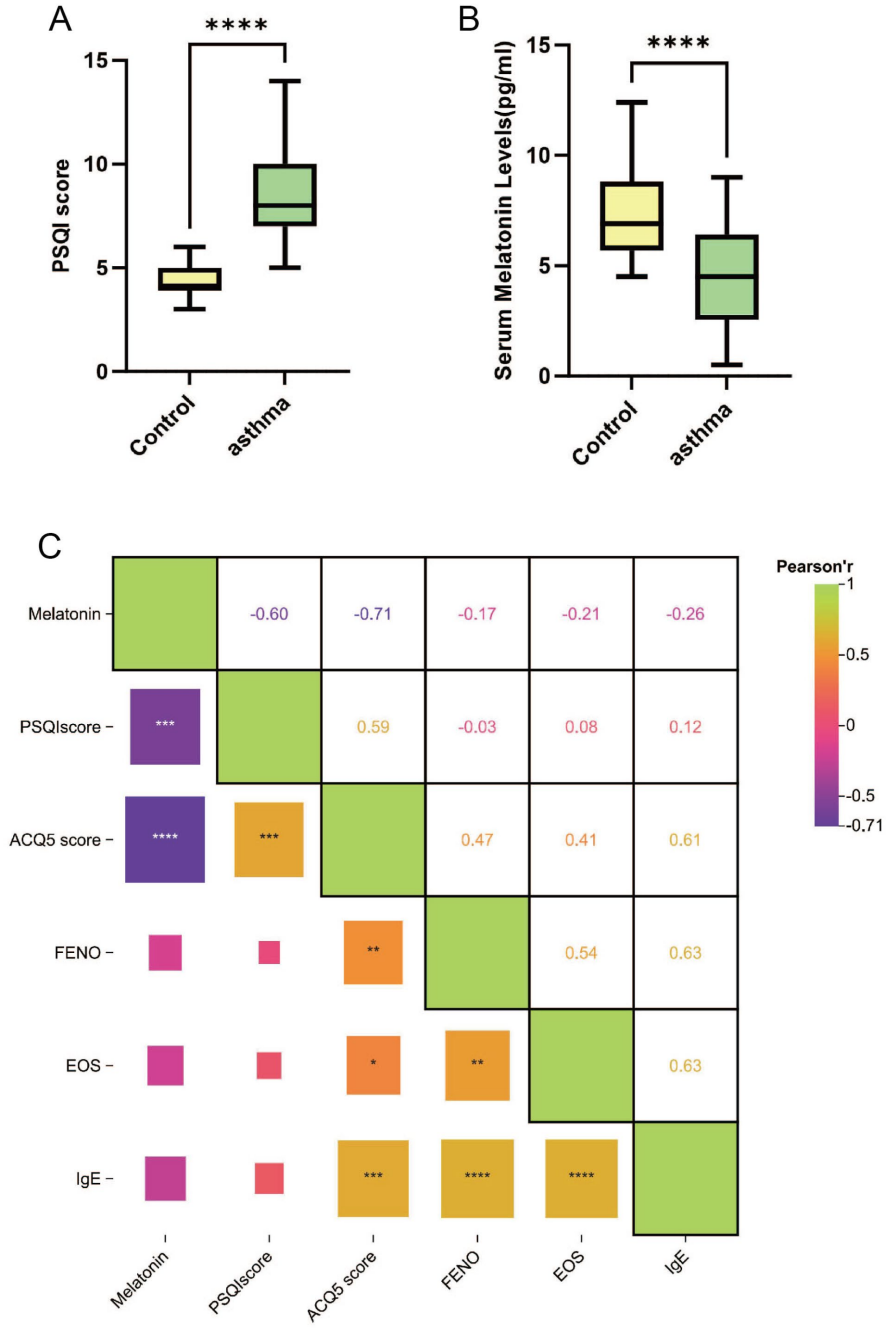
Comparison of data between asthma patients and the control group, and within the asthma group. **(A)** Comparison of serum melatonin levels between the asthma group and the control group. **(B)** Comparison of serum cortisol levels between the asthma group and the control group. **(C)** Pearson correlation analysis of various indicators within the asthma group. *p < 0.05, **p < 0.01, ***p < 0.001, ****p < 0.0001.

A total of 33 patients with asthma and 25 healthy individuals participated in the study, completing the questionnaires. No statistically significant differences were observed in age, gender, or BMI between the two groups. We compared lung function, Pittsburgh Sleep Quality Index (PSQI) scores, melatonin levels, and cortisol levels between the groups. The results revealed significant differences in all measured indicators, with statistical significance set at *p* < 0.05. These findings suggest that the observed differences in physiological indicators between the two groups may be associated with the factors under investigation. Detailed results are presented in Table 1 and Figures 2A,B.

**Table 1:** Baseline characteristics of patients in the control and asthma groups: a comparative analysis.

Group	*N*	Sex (M/F)	Age (years)	BMI (kg/m^2^)	PSQI score	Cortisol (nmol/L)	Melatonin (pg/mL)	Lung function		
								FEV1 improvement rate (%)	FEV1% pred (post-medication)	FEV1/FVC (post-medication)
Control	25	11/14	34.67 ± 9.13	24.55 ± 2.88	4.52 ± 0.92	117.90 ± 35.00	7.34 ± 2.15	2.30 ± 2.42	97.83 ± 10.48	85.42 ± 6.70
Asthma	33	16/17	30.49 ± 8.73	26.01 ± 5.20	8.58 ± 2.05	251.33 ± 67.40	4.51 ± 2.36	23.33 ± 21.50	79 ± 20.85	74.06 ± 12.20
*t*/*X*^2^ value		0.115	1.75	−1.15	−9.99	−9.78	4.70	−5.573	4.470	4.438
*p*-value		0.735	0.085	0.258	0.000	0.000	0.000	0.000	0.000	0.000

Data are expressed as mean ± SEM.

### 3.2 Data on asthma patients

All asthma patients in the study exhibited poor sleep quality (PSQI > 5) (17). Cortisol levels were significantly elevated compared to the control group, while melatonin levels were significantly lower, with both differences reaching statistical significance. Within the asthma group, all indicators followed a normal distribution, allowing for Pearson correlation analysis. The results revealed significant correlations between asthma control levels and PSQI scores, IgE, melatonin levels, eosinophils in peripheral blood and fractional exhaled nitric oxide (FeNO), all showing statistical significance. Detailed results are provided in Figure 2C and Supplementary Table S1.

### 3.3 Induction of allergic airway inflammation successfully achieved with OVA

**Figure 3:**
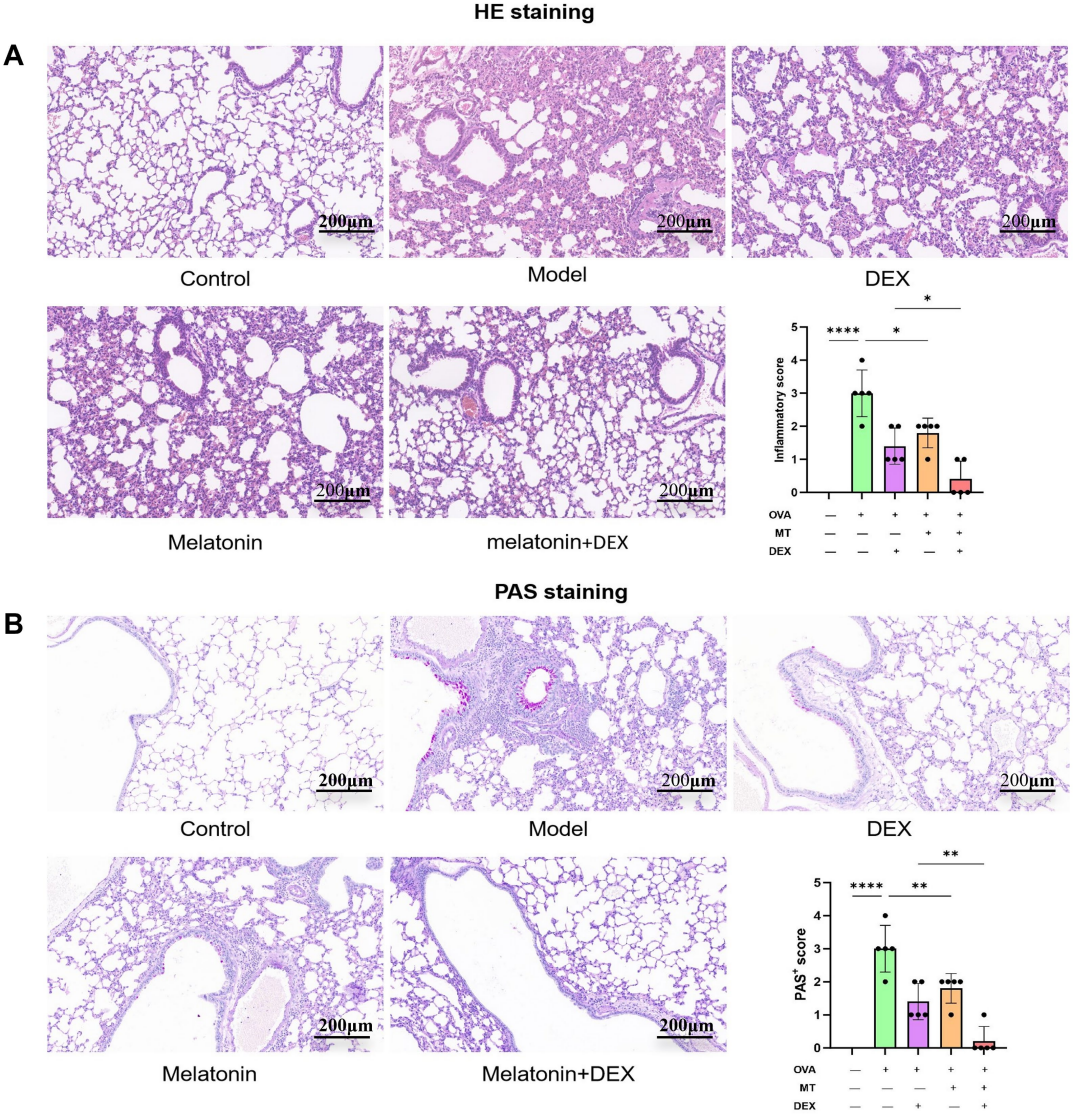
Histopathological analysis of mouse lung tissue. **(A)** H&E staining (×200), with inflammation quantified based on H&E staining. **(B)** PAS staining (×200), with quantification of PAS staining. *p <0.05, **p <0.01, ****p <0.0001.

**Figure 4:**
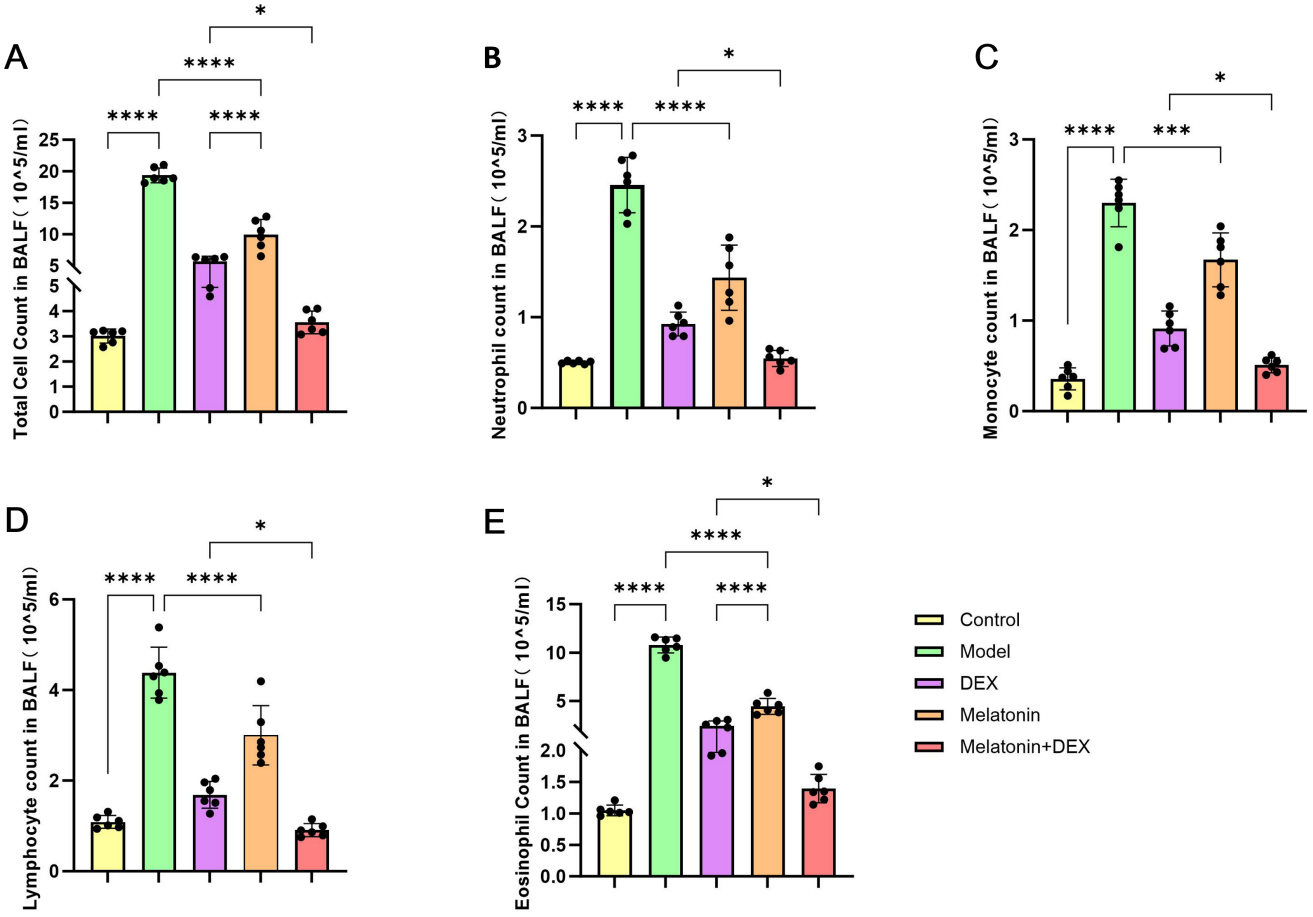
Cytological analysis of mouse bronchoalveolar lavage fluid. **(A)** Total cell count in BALF. **(B)** Neutrophil count in BALF. **(C)** Monocyte count in BALF. **(D)** Lymphocyte count in BALF. **(E)** Eosinophil count in BALF. *p < 0.05, ***p < 0.001, ****p < 0.0001.

**Figure 5:**
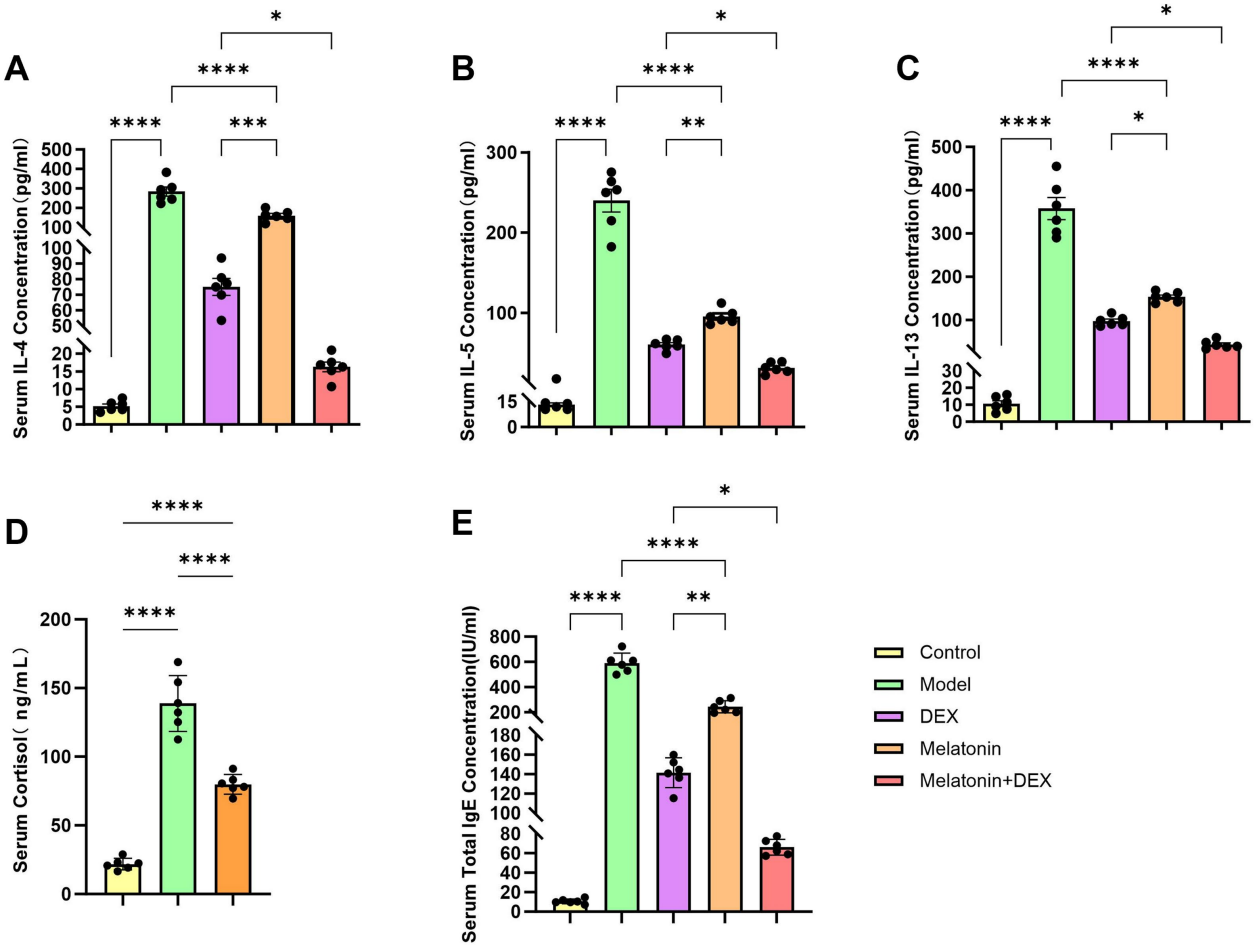
Analysis of serum biomarkers in mice. **(A)** Serum IL-4 concentration. **(B)** Serum IL-5 concentration. **(C)** Serum IL-13 concentration. **(D)** Serum cortisol concentration. **(E)** Serum total IgE concentration. *p < 0.05, **p < 0.01, ***p < 0.001, ****p < 0.0001.

**Figure 6:**
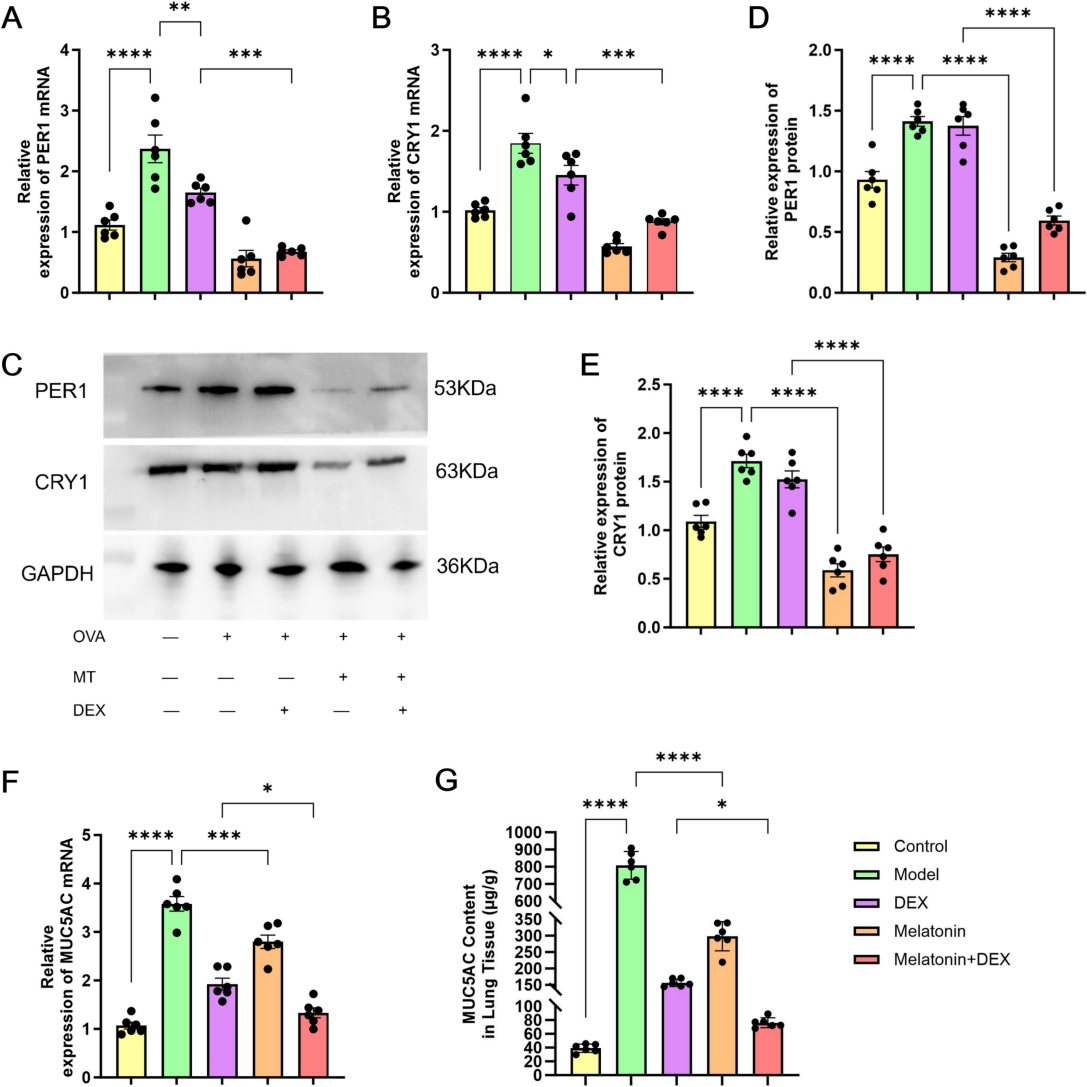
Expression of biomarkers in mouse lung tissue. **(A,B)** Relative mRNA expression levels of PER1 and CRY1 measured by qPCR. **(C–E)** Protein expression of PER1 and CRY1 measured by western blot. **(F)** Relative mRNA expression level of MUC5AC measured by qPCR. **(G)** MUC5AC protein levels in lung tissue measured by ELISA. *p < 0.05, **p < 0.01, ***p < 0.001, ****p < 0.0001.

To establish a model of allergic airway inflammation, specific pathogen-free (SPF) BALB/c mice were employed (18). In contrast to the control group, the administration of 1% OVA led to notable infiltration of inflammatory cells within the airways and increased goblet cell hyperplasia, as revealed by hematoxylin and eosin (H&E) staining (Figure 3A) and periodic acid-Schiff (PAS) staining (Figure 3B). Additionally, the OVA challenge resulted in a considerable rise in the overall cell population in bronchoalveolar lavage fluid (BALF), encompassing eosinophils, monocytes, lymphocytes, and neutrophils, with eosinophils being particularly elevated (Figure 4). ELISA analysis demonstrated marked increases in total IgE and MUC5AC levels (Figures 5F, 6D), accompanying this was an increase in the production of Th2-associated cytokines (IL-4, IL-5, and IL-13) (19) when compared to the control group (Figures 5A–C).

### 3.4 Amelioration of allergic airway inflammation by melatonin supplementation

To investigate the therapeutic effects of melatonin on OVA-induced allergic airway inflammation in mice, exogenous melatonin was administered to intervene in the model. HE and PAS staining showed that cellular infiltration and mucus secretion were significantly alleviated in the melatonin-treated group compared to the model group (Figure 3). Inflammatory cell levels in bronchoalveolar lavage fluid (BALF) were markedly reduced (Figure 4), and serum levels of type 2 inflammatory cytokines, including IL-4, IL-5, IL-13, and IgE, were significantly lower in the melatonin-treated group than in the model group (Figures 5A–C,E). All these differences between the two groups were statistically significant.

### 3.5 Increased corticosterone expression

Subsequently, we assessed the expression levels of cortisol in serum samples (Figure 5D). Our findings indicated that serum corticosterone levels were markedly elevated in the model group compared to the control group. This elevation suggests that type 2 airway inflammation may be associated with glucocorticoid tolerance or resistance, highlighting the urgent need for improved or combined therapeutic strategies to achieve better disease control.

### 3.6 Melatonin and glucocorticoid co-therapy reduces pathological damage, mucus secretion, and inflammation

In Figure 3, it is evident that the HE staining reveals significantly reduced pathological damage and PAS-positive areas in the Melatonin + DEX group compared to the DEX or Melatonin monotherapy group. Asthma is characterized by a complex network of inflammatory responses involving cytokines and IgE (20, 21), with IL-4, IL-5, and IL-13 serving as key cytokines that drive pathogenic Th2 responses in allergic airway disease (22). Consequently, we assessed serum levels of IgE, IL-4, IL-5, and IL-13. As illustrated in Figures 5A–C, E, levels of IL-4, IL-5, IL-13, and IgE were significantly elevated in the model group relative to the control group. Treatment with both dexamethasone and melatonin led to a reduction in the levels of IgE, IL-4, IL-5, and IL-13, with the most pronounced decrease occurring in the group receiving melatonin in conjunction with glucocorticoids. Additionally, we evaluated cell counts and classifications in bronchoalveolar lavage fluid, which exhibited significant reductions following treatment with either dexamethasone or melatonin, with the most substantial decrease observed in the group receiving dexamethasone combined with melatonin (Figure 4).

### 3.7 Expression analysis of PER1/CRY1 gene and MUC5AC

The QPCR was used to quantify MUC5AC mRNA expression, and ELISA was performed to measure MUC5AC protein levels (Figures 6F,G) in lung tissues showed a significant increase in the model group compared to controls. Treatment with dexamethasone and melatonin significantly reduced MUC5AC levels in the lung tissues of mice, with statistically significant differences from the model group. The combination of glucocorticoids and melatonin resulted in the greatest reduction, enhancing the effect compared to single treatments. To assess changes in peripheral lung PER1 and CRY1 expression associated with OVA-induced allergic airway inflammation, we performed quantitative PCR (qPCR) and Western blot analyses to assess the mRNA and protein expression levels of the circadian rhythm–related genes PER1 and CRY1 in lung tissues. Our findings revealed a significant upregulation of PER1 (Figures 6A,C,D) and CRY1 (Figures 6B,C,E) in response to OVA exposure, suggesting that the circadian regulation in the lungs is disrupted during the course of allergic airway inflammation. In groups treated with melatonin or glucocorticoids, expression levels of PER1 and CRY1 genes in lung tissues were significantly reduced compared to the model group, with the most substantial decrease observed in the melatonin treatment group. The reduction in PER1 and CRY1 mRNA and proteins expression was more pronounced in the melatonin combined with glucocorticoid treatment group than in the glucocorticoid-only group, although it remained less than that in the melatonin-only treatment group.

## 4 Discussion

Despite advances in asthma therapy, a subset of patients with allergic asthma remains inadequately controlled, highlighting the need for adjunctive strategies beyond conventional pharmacological approach (20–24). In this study, we investigated the associations between melatonin levels, circadian rhythm regulation, asthma control, and sleep quality in patients with allergic asthma, and further explored the effects of melatonin, alone and in combination with corticosteroids, on airway inflammation and circadian gene expression using an experimental mouse model.

Recent studies have highlighted the role of circadian rhythm disruption in asthma severity and disease progression (25). Consistent with these reports, our findings demonstrate significant associations between asthma control, sleep quality, and circadian-related hormonal profiles, supporting the clinical relevance of circadian regulation in allergic asthma. Circadian rhythms play a critical role in modulating immune responses, and disruption of these rhythms has been linked to worsening asthma symptoms (26, 27). Moreover, restoration of circadian rhythm stability has been reported to improve therapeutic outcomes in allergic diseases (28), underscoring circadian regulation as a potential therapeutic target supported by both recent literature and our data.

Melatonin exerts cytoprotective effects by enhancing antioxidant defenses, reducing lipid peroxidation, suppressing inflammatory mediators, and inhibiting apoptosis, leading to preservation of dopaminergic neurons in experimental models of Parkinson’s disease (29, 30). Pre-treatment or prolonged administration appears more effective, highlighting its therapeutic potential. These biological effects provide a rationale for the adjunctive use of melatonin with glucocorticoids in allergic airway diseases. Beyond these protective mechanisms, melatonin also regulates sleep–wake cycles and circadian rhythm homeostasis, with levels rising in the evening to promote sleep and falling in the morning to signal wakefulness (31, 32). Disruptions in circadian rhythms may exacerbate asthma symptoms, while asthma itself may impair melatonin secretion, suggesting a bidirectional relationship (33). Our findings support the concept that stabilizing circadian rhythms through melatonin supplementation could offer additional therapeutic benefit in allergic asthma.

Corticosteroids are the cornerstone of asthma therapy, effectively controlling inflammation at various disease severities. However, their long-term use can disrupt circadian rhythm stability, which may have negative implications for asthma control. The hypothalamic–pituitary–adrenal (HPA) axis, which regulates the secretion of cortisol, is itself governed by circadian rhythms. Disruptions to this rhythm can influence the HPA axis and potentially worsen asthma symptoms (34, 35). In this context, our results suggest that melatonin may serve as an adjunctive therapy capable of stabilizing circadian rhythms while enhancing the anti-inflammatory efficacy of corticosteroids.

In this study, we examined the associations between melatonin levels, sleep quality, and asthma control by comparing patients with allergic asthma and matched healthy controls. As expected, the asthma group demonstrated a greater bronchodilator response and higher levels of blood eosinophils, FeNO, and serum IgE, confirming the allergic asthma phenotype. Significant differences were also observed in PSQI scores, melatonin, and cortisol levels between the two groups, suggesting that asthma is accompanied by alterations in sleep quality and circadian-related hormonal profiles. Correlation analyses within the asthma group showed that poorer asthma control was associated with higher PSQI scores, IgE levels, eosinophil counts, and cortisol concentrations, as well as lower melatonin levels. Sleep quality was positively correlated with cortisol and inversely correlated with melatonin. Given the relatively small sample size, the case–control design, and the potential influence of unmeasured clinical and sleep-related factors, the observed associations should be interpreted with caution, and causality cannot be inferred. Nevertheless, the inverse association between melatonin and cortisol supports a dynamic interplay between circadian hormones that may influence both sleep quality and asthma control.

To investigate the potential therapeutic effects of melatonin further, we established an allergic airway inflammation model in Balb/c mice using ovalbumin (OVA). The success of the model was confirmed by the increased expression of eosinophils in bronchoalveolar lavage fluid (BALF) and elevated levels of IL-4, IL-5, IL-13, and IgE in the serum. Melatonin, corticosteroids, and their combination were administered to the allergic asthma model to evaluate their impact on type 2 inflammation. The results demonstrated that both melatonin and corticosteroid treatments significantly reduced type 2 inflammatory markers in the mice, compared to the control group, with statistically significant differences. This suggests that melatonin has a modulating effect on the expression of type 2 inflammatory cytokines and inflammatory cells in BALF. Furthermore, the combination of melatonin and corticosteroids was found to have the most pronounced effect, significantly reducing the levels of inflammatory cytokines, inflammatory cells in BALF, and MUC5AC expression compared to either treatment alone. These results suggest a synergistic anti-inflammatory effect of melatonin and corticosteroids. Moreover, we observed that serum levels of cortisol and melatonin exhibited an interplay, which may be key to understanding the therapeutic potential of melatonin in asthma. Investigating circadian rhythm genes, we found that melatonin treatment helped stabilize the expression of PER1 and CRY1 genes, which play pivotal roles in maintaining circadian rhythm stability. The combination of melatonin and corticosteroids was more effective in preserving circadian rhythm stability than corticosteroid treatment alone. These findings suggest that melatonin may enhance the anti-inflammatory effects of corticosteroids by modulating circadian rhythms.

This study provides important insights into the potential role of melatonin in enhancing corticosteroid therapy for asthma, particularly through the regulation of circadian rhythms. The results support the hypothesis that melatonin, through its ability to stabilize circadian rhythms, may help optimize corticosteroid efficacy, leading to improved asthma control and better patient outcomes.

The limitations of this study should be noted. The relatively small sample size and lack of long-term follow-up may limit the generalizability and evaluation of sustained melatonin efficacy. Samples were collected at a single time point, which may not fully capture the circadian dynamics of inflammatory factors, cortisol, and melatonin, though this allowed standardized comparisons among groups. While our results support a role for melatonin in allergic airway inflammation, formal mechanistic experiments were not performed. Although the results suggest additive effects of melatonin and glucocorticoids on inflammatory markers, formal pharmacological analyses were not conducted to confirm true synergy. Future studies with larger cohorts, longitudinal follow-up, multi-time-point sampling, and targeted mechanistic and synergy analyses will help validate and extend these findings.

## 5 Conclusion

This study reveals the synergistic interactions between melatonin and glucocorticoids in alleviating allergic airway inflammation, underscoring their potential application in asthma management. Our findings demonstrate a significant correlation between melatonin levels, asthma control scores, and sleep quality. Furthermore, in a mouse model, melatonin treatment significantly reduced inflammatory cell infiltration in the airways and altered the expression of the circadian rhythm-associated genes PER1 and CRY1, particularly when combined with glucocorticoids, indicating a notable synergistic effect.

Thus, melatonin emerges as a promising therapeutic candidate for allergic airway diseases, presenting a novel approach to asthma treatment strategies. Interventions aimed at regulating circadian rhythms may improve clinical outcomes for asthma patients. However, further research is necessary to elucidate the underlying mechanisms and long-term efficacy of melatonin across diverse asthma patient populations. We recommend that future studies focus on the clinical application of melatonin to optimize the management and treatment of allergic asthma.

## Data Availability

The original contributions presented in the study are included in the article/Supplementary material, further inquiries can be directed to the corresponding authors.
