# Correction: Synergistic interactions of melatonin and glucocorticoids in alleviating allergic airway inflammation

**DOI:** 10.3389/fmed.2026.1818337

**Published:** 2026-03-13

**Authors:** Zhe Zhang, Jipeng Wang, Ting Ma, Xiuqin Zhang, Guilian Chen, Baolan Wang

**Affiliations:** 1 Physical Examination Center, The Affiliated Huai'an No.1 People's Hospital of Nanjing Medical University, Huai'an, Jiangsu, China; 2 Department of Respiratory Medicine, The Huai'an Clinical College of Xuzhou Medical University, Huai'an, Jiangsu, China; 3 Department of Respiration Medicine, The First Affiliated Hospital of Soochow University, Suzhou, Jiangsu, China

**Keywords:** allergic diseases, asthma, circadian rhythm, glucocorticoids therapy, melatonin

There was a mistake in the caption of “**Figure 5**” as published. The item “(E) Serum melatonin concentration” was mistakenly included in the figure legend, and Panel E was incorrectly described. The corrected caption of “**Figure 5**” appears below:

“**Figure 5**. Analysis of serum biomarkers in mice. **(A)** Serum IL-4 concentration. **(B)** Serum IL-5 concentration. **(C)** Serum IL-13 concentration. **(D)** Serum cortisol concentration. **(E)** Serum total IgE concentration. ^*^p < 0.05, ^**^p < 0.01, ^***^p < 0.001, ^****^p < 0.0001.”

There was a mistake in the in-text citation as published. The citation “**Figures 5A–C, F**” should be corrected to “**Figures 5A–C, E**”.

A correction has been made to the section “**3 Results**, subsection *3.6 Melatonin and glucocorticoid co-therapy reduces pathological damage, mucus secretion, and inflammation*, Paragraph 1”:

“As illustrated in **Figures 5A–C, E**, levels of IL-4, IL-5, IL-13, and IgE were significantly elevated in the model group relative to the control group”.

The original version of this article has been updated.

