# Supplementary material for: Synergistic interactions of melatonin and glucocorticoids in alleviating allergic airway inflammation
Source: Front Med (Lausanne). 2026 Feb 25;13:1738965. doi: 10.3389/fmed.2026.1738965 (PMC12977073; doi:10.3389/fmed.2026.1738965)
Supplement: Supplementary file 1 [file Table_1.docx]

Table S1 Pearson correlation analysis among indicators in asthma patients

|  | FENO | ACQ5 score | PSQI score | EOS | IgE | Melatonin |
| --- | --- | --- | --- | --- | --- | --- |
| FENO | 1 |  |  |  |  |  |
| ACQ5 score | 0.474** | 1 |  |  |  |  |
| PSQI score | -0.034 | 0.590** | 1 |  |  |  |
| EOS | 0.538** | 0.413* | 0.075 | 1 |  |  |
| IgE | 0.634** | 0.612** | 0.124 | 0.634** | 1 |  |
| Melatonin | -0.172 | -0.712** | -0.601** | -0.210 | -0.262 | 1 |

*P<0.05, **P<0.05, ***P<0.001, ****P<0.0001.
